# Spontaneous formation of a chiral (Mo_2_O_2_S_2_)^2+^-based cluster driven by dimeric {Te_2_O_6_}-based templates†
†Electronic supplementary information (ESI) available: additional synthesis details, analytical data and crystallographic data. CSD 433070, 433071 and CCDC 1550063. For ESI and crystallographic data in CIF or other electronic format see DOI: 10.1039/c8dt00832a


**DOI:** 10.1039/c8dt00832a

**Published:** 2018-03-29

**Authors:** Jamie W. Purcell, De-Liang Long, Edward C. Lee, Leroy Cronin, Haralampos N. Miras

**Affiliations:** a WestCHEM , School of Chemistry , University of Glasgow , Glasgow , G12 8QQ , UK . Email: Charalampos.moiras@glasgow.ac.uk ; Email: Lee.cronin@glasgow.ac.uk

## Abstract

Utilization of [Mo_2_S_2_O_2_(H_2_O)_6_]^2+^ and a tellurite anion led to the formation of three new clusters, **1–3**, with unique structural features. The tellurite anion not only templated the formation of [(Mo_2_O_2_S_2_)_4_(TeO_3_)(OH)_9_]^3–^**1** and [(Mo_2_O_2_S_2_)_12_(TeO_3_)_4_(TeO_4_)_2_ (OH)_18_]^10–^**3**, but also the *in situ* generation of two different types of dimeric {Te_2_O_6_} based moieties induced the spontaneous assembly of the chiral [(Mo_2_O_2_S_2_)_10_(TeO_3_)(Te_2_O_6_)_2_(OH)_18_]^8–^ anionic cluster, **2**.

## Abstract 2

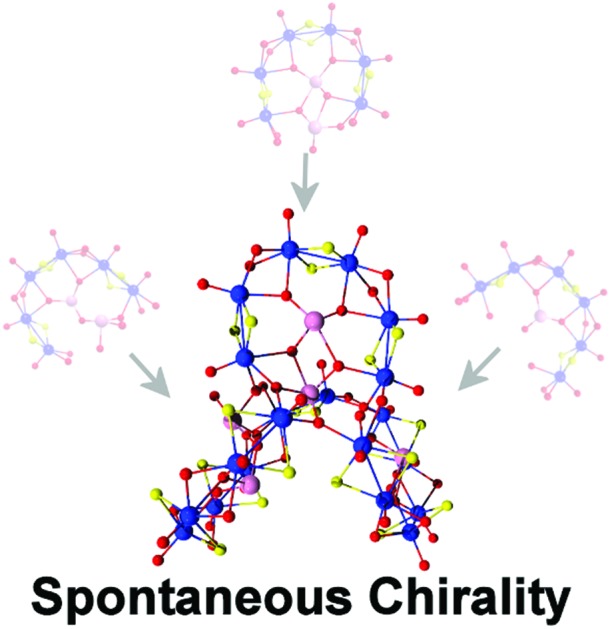
Utilization of tellurite anion as template, led to the formation of unprecedented oxothiometalate based building blocks and the spontaneous manifestation of chirality.

Polyoxometalates (POMs) are metal oxide-based molecular clusters that have attracted a lot of attention in the last three decades due to their structural versatility^1^–^3^ and the wide variety of applications in which they have found use, including medicine,^4^ electronics^5^ and catalysis.^6^ Polyoxothiometalates (POTMs) are a “softer” POM subset of inorganic clusters which incorporate sulfur atoms. These materials constitute a molecular equivalent of bulk metal chalcogenide materials, in the same way that conventional POMs can be considered as molecular fragments of bulk metal oxides. Thus, the numerous functionalities displayed by chalcogenide based materials^7^–^9^ can be potentially coupled with the structural diversity and design principles of POM chemistry in order to form novel materials tailored for specific applications.^10^–^12^

The principal factor that distinguishes POTMs from conventional POMs is the unique set of building blocks from which these compounds are derived. Whereas conventional POMs are synthesised in most cases directly from mononuclear metal oxide anions (most commonly MoO_4_^2–^ or WO_4_^2–^),^13^–^16^ POTMs generally require the preparation of multinuclear building blocks that can be used as starting materials in synthesis procedures followed by their condensation at elevated pH values in marked contrast to POM chemistry.^17^,^18^ A common building block is the dinuclear cation [Mo_2_O_2_S_2_]^2+^ which forms the basis of the work reported herein.^19^–^22^

In the absence of any other structure directing agents, the structures formed by the condensation of the [Mo_2_O_2_S_2_]^2+^ dimer are limited to ring shaped clusters of limited nuclearity.^23^ However, the incorporation of additional metal centres can be achieved in the presence of templating agents such as carboxylate anions with multiple carboxylic groups, as demonstrated in previous work by Cadot *et al.*^24^–^26^ In order to deviate substantially from the observed topologies and specific range of nuclearities, it was necessary to generate new building block libraries of sufficient diversity in terms of the number of available constituents which can readily assemble into larger species. This was achieved by the introduction of templates of appropriate size and sufficient rigidity leading to the formation of new building blocks with unsaturated coordination sites which can readily assemble into high nuclearity architectures and give rise to interesting structural features. The combination of the organic squarate (C_4_O_4_^2–^)^27^ with selenite (SeO_3_^2–^) or tellurite (TeO_3_^2–^) anions^28^ is a representative example of templates that facilitated the generation of new open-ring shaped building blocks leading to an unprecedented number of new compounds with unprecedented structural features^29^–^31^ and generation of spontaneous chirality.^30^

While it has been demonstrated that the selenite anion can template constructively [Mo_2_O_2_S_2_]^2+^ cationic dimers and form intricate architectures, it has been the only inorganic species observed to template the formation of building blocks on their own. In an effort to expand and diversify the existing building block library, it was hypothesised that the larger atomic radius of the tellurite anion, TeO_3_^2–^, its tendency for aggregation and difference in redox properties^32^–^34^ compared to SeO_3_^2–^ anions could influence the assembly process and potentially trigger redox processes which could lead to the generation of new building block libraries and facilitate the formation of new species.

Herein, we demonstrate the efficacy of the tellurite anion to act as a template and trigger the formation of new building block libraries. We report the discovery of three new clusters: K_3_[(Mo_2_O_2_S_2_)_4_(TeO_3_)(OH)_9_]·20H_2_O (**1**), K_8_[(Mo_2_O_2_S_2_)_10_(TeO_3_) (Te_2_O_6_)_2_(OH)_18_]·45H_2_O (**2**) and (C_4_H_12_N)K_9_[(Mo_2_O_2_S_2_)_12_(TeO_3_)_4_ (TeO_4_)_2_(OH)_18_]·48H_2_O (**3**) (Fig. 1). Interestingly, the higher tendency of the tellurite anion for aggregation led to the *in situ* formation of two different types of dimeric {Te_2_O_6_} templates leading to the spontaneous formation of a chiral POTM cluster, **2**.

**Fig. 1:**
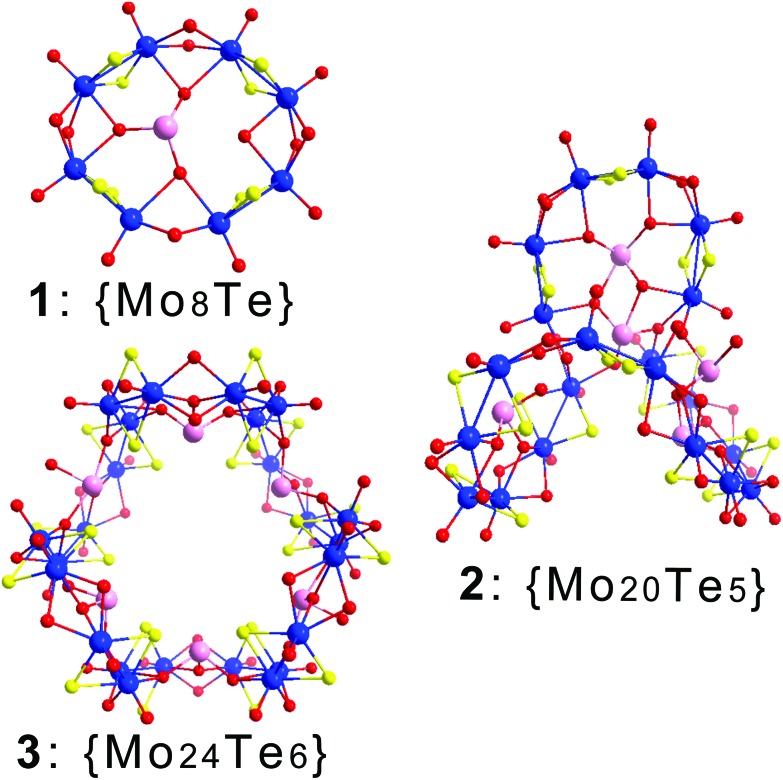
Structural representations of three new Te-templated POTMs: **1**, [(Mo_2_O_2_S_2_)_4_(TeO_3_)(OH)_9_]^3–^; **2**, [(Mo_2_O_2_S_2_)_10_(TeO_3_) (Te_2_O_6_)_2_(OH)_18_]^8–^; **3**, [(Mo_2_O_2_S_2_)_12_(TeO_3_)_4_(TeO_4_)_2_(OH)_18_]^10–^.

The presence of the tellurite anion in the [Mo_2_O_2_S_2_]^2+^ solution gave rise to a series of new building blocks (Fig. 2). The building blocks **A** and **B** are two “open-ring” moieties consisting of two and three [Mo_2_O_2_S_2_]^2+^ units which are templated by a pyramidal tellurite anion exhibiting two types of μ_4_-coordination modes, η^2^:η^1^:η^1^–O:O:O and η^2^:η^2^–O:O, respectively. The **A** and **B** building blocks are isostructural to the selenium and tellurium templated ones which have been reported by our group previously.^28^,^31^ The characteristic bond lengths observed for the two building blocks can be summarized as follows: Te–O bond lengths for **A** and **B** vary from 1.887(14)–1.921(9) and 1.831(8)–1.865(7) Å; Mo–Mo bond lengths within the [Mo_2_O_2_S_2_]^2+^ unit are found to be 2.831(1)–2.866(1) and 2.807(1)–2.848(1) Å; Mo–S bond lengths vary from 2.231(3)–2.360(3) and 2.306(3)–2.344(3) Å; and Mo=O bond lengths vary from 1.664(8)–1.695(7) and 1.666(9)–1.693(8) Å, respectively. Finally, the Mo–O(Te) distances are found to be 2.142(7)–2.499(8) and 2.198(7)–2.373(7) Å while the distance between the Mo centres and the hydroxo bridges Mo–O(H) spans the ranges 2.056(7)–2.171(7) and 2.100(8)–2.149(8) Å.

**Fig. 2:**
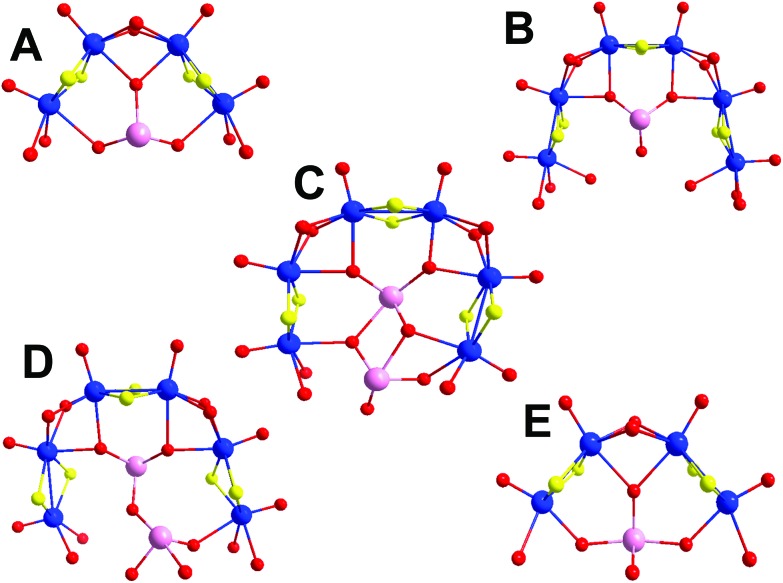
Structural representations of the identified building blocks: **A**, [(Mo_2_O_2_S_2_)_2_(TeO_3_)(OH)_6_]^4–^; **B**, [(Mo_2_O_2_S_2_)_3_(TeO_3_)(OH)_10_]^6–^; **C**, [(Mo_2_O_2_S_2_)_3_(Te_2_O_6_)(OH)_8_]^4–^; **D**, [(Mo_2_O_2_S_2_)_3_(Te_2_O_6_)(OH)_8_]^4–^; and **E**, [(Mo_2_O_2_S_2_)_2_(TeO_4_)(OH)_6_]^4–^. Colour code: Mo, blue; O, red; S, yellow; and Te, pink.

Interestingly, the interaction of tellurite anions with the [Mo_2_O_2_S_2_]^2+^ dimer units gave rise to the formation of two fundamentally new building blocks, [(Mo_2_O_2_S_2_)_3_(Te_2_O_6_)(OH)_8_]^4–^**C** and **D**. The discovery of these two new building blocks was possible due to the ability of the tellurite anion to aggregate into small clusters which can then template further the formation of [Mo_2_O_2_S_2_]^2+^ building blocks. This chemical behaviour has been observed before where small multi-nuclear tellurite fragments were trapped by vanadium-based polyoxometalate fragments as reported by Norquist *et al.*, although organic species have been known to trap these types of moieties as well.^35^–^38^ Even though **C** and **D** building blocks might look similar, there is a crucial difference of the connectivity between the tellurium atoms. In **C**, the tellurium atoms are linked through two bridging oxygen atoms with varying Te–O bond lengths, 1.924(6)–2.270(7) Å while building block D exhibits only a single bridging oxygen atom between the two tellurium atoms with the relevant Te–O bond lengths falling within the range of 1.872(6) and 2.220(6) Å. The difference in connectivity which is reflected in the bond distances, and causes significant distortion in the “open-ring” structure and asymmetry in the available coordination sites is proven to be crucial during the assembly process, which will be discussed below.

Finally, building block **E** is very similar to **A**; however, the tellurite centre is four coordinated in this case forming a [Te^IV^O_4_]^4–^ anion. It is important to note here that the observed change in coordination mode is not associated with a change in its oxidation state as shown by bond valence sum (BVS) calculations. The [Te^IV^O_4_]^4–^ template adopts a “see-saw” configuration; the two “arm” oxygen atoms display an angle of 163.2(3)° while the “pivot” oxygen atoms display an angle of 98.6(4)°. The “arm” oxygen atoms are coordinated to one Mo-centre each, where one of the “pivot” oxygen atoms is coordinated to two and the other one is free. In this case, the Te–O bonds appeared to be elongated as expected; the Te–O_(arm)_ bond lengths fall in the range 2.034(9)–2.042(9) Å, while the Te–O_(pivot)_ (coordinated) and Te–O_(pivot)_ (uncoordinated) bonds were found to be 1.864(9) and 1.940(10) Å, respectively. Finally, the rest of the bond distances appeared to be in agreement with the previously discussed **A–D** building blocks; the Mo–Mo, Mo–S and Mo=O distances were found to be 2.819(1)–2.826(1), 2.315(3)–2.334(3) and 1.683(9)–1.701(9) Å, respectively. Finally, the bonding distances between the oxygen of the template and the Mo centres range from 2.142(8)–2.155(9) Å [Te–O_(arm)_] and 2.290(9)–2.297(9) Å [Te–O_(pivot)_].

Interestingly, the existence of the **A–E** building blocks in the reaction mixtures became evident also during the course of the electrospray ionization mass spectrometry (ESI-MS) studies in solution. It was possible to identify not only the intact **1–3** species but also their building blocks generated by partial fragmentation of the clusters into their most stable components (see the ESI†).

The first of the newly discovered compounds, **1**, is an 8-membered, asymmetrical molybdenum ring templated by a single tellurite anion positioned in an off-centre position due to its trigonal pyramidal geometry. It consists of one **A**-type building block connected through hydroxide bridges to two additional dimer units that complete the ring-shaped structure (Fig. 3). The point group of this molecule is C_S_, indicating that the only significant symmetry element is a reflection plane which includes the tellurium atom and one Te–O bond and is perpendicular to the plane of the ring. The asymmetric templation of the ring by the tellurite anion reduced significantly the symmetry elements of the structure which in similar cases usually exhibits a 3- or even 4-fold axis.

**Fig. 3:**
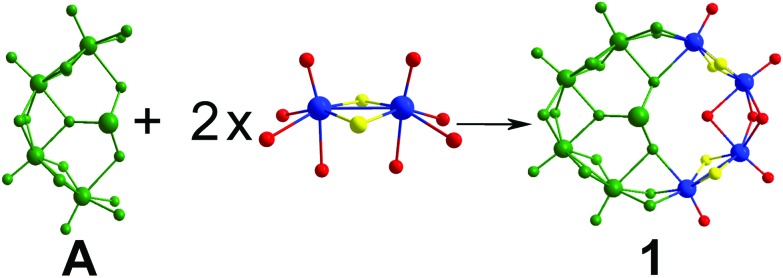
Structural representation of the formation of **1** from an **A**-type building block and two additional [Mo_2_O_2_S_2_]^2+^ dimer units.

Compound **2** is best described as having a pseudo-trigonal “twist” shape. It is constructed using three different types of building blocks, **B**, **C** and **D**, with an additional dimeric unit serving as a linker between the **B** and **D** building blocks (Fig. 4). As noted earlier, the *in situ* generation of two different types of {Te_2_O_6_} templates induces asymmetry and structural distortion to the building blocks **C** and **D** which leads to the unusual topology of cluster **2** as the building blocks need to be oriented in such a way as to allow the most energetically favourable orientation between them. Interestingly, the above factors led to the spontaneous formation of the chiral cluster **2** with a C_1_ point group. Ultimately, the cluster forms a racemic unit cell and a centrosymmetric space group, *P*1.

**Fig. 4:**
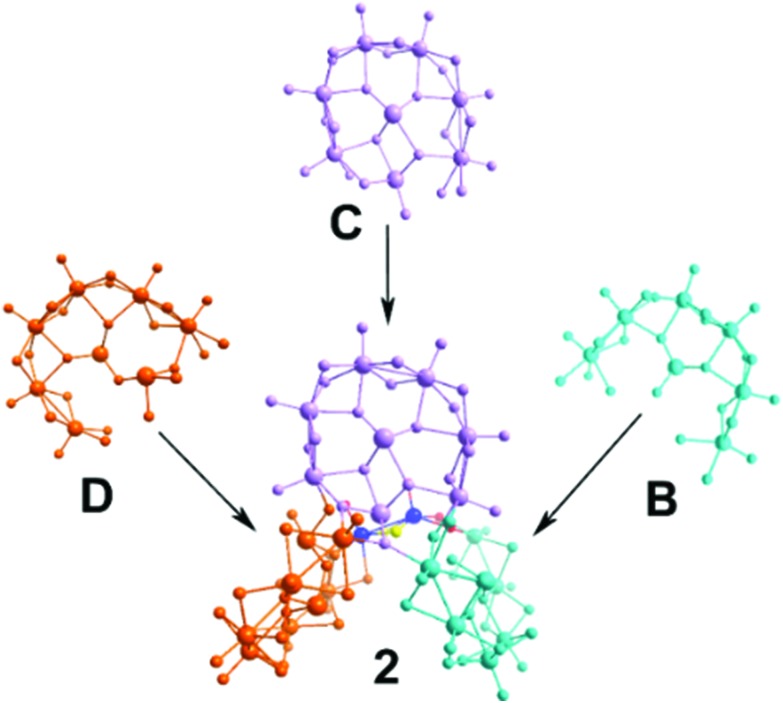
Structural representation of **2** highlighting the connectivity of the building blocks present in **2** (**B**, cyan; **C**, lavender; and **D**, orange). Mo, blue spheres; S, yellow spheres.

An alternative way to visualize the structure of **2** and understand better the connectivity and the relative orientation between the building blocks is shown in Fig. 5. Each building block is defined by a plane and forms a specific dihedral angle with its neighbouring one. The plane defined by the **D** building block – denoted in orange in Fig. 5 – forms an angle of 72.88° with the plane defined by the **C** building block (blue) and 60.45° to the **B** (red plane in Fig. 6). The **B** and **C** building blocks form an angle of 67.21° to each other.

**Fig. 5:**
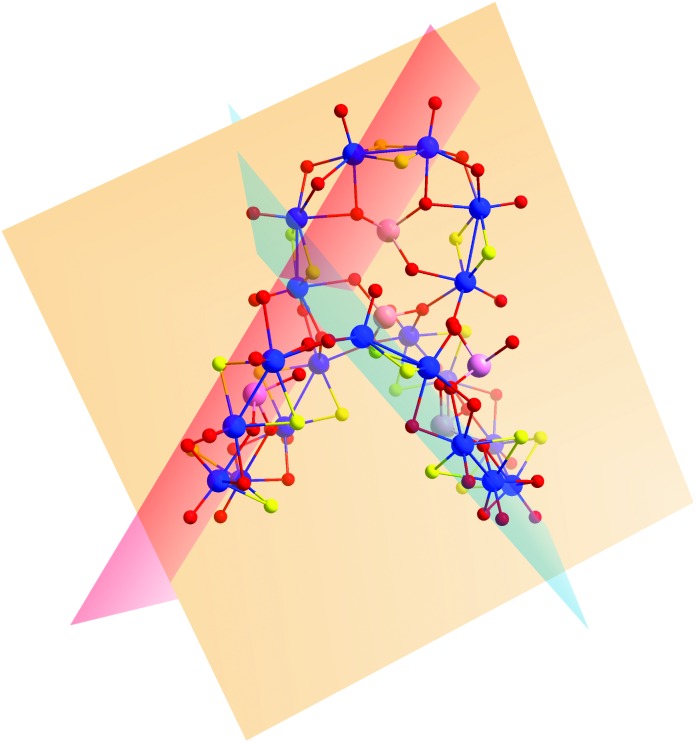
A schematic showing the planes defined by each of the building blocks in compound **2** – building block **D** represented in orange, **C** in blue and **B** in red.

**Fig. 6:**
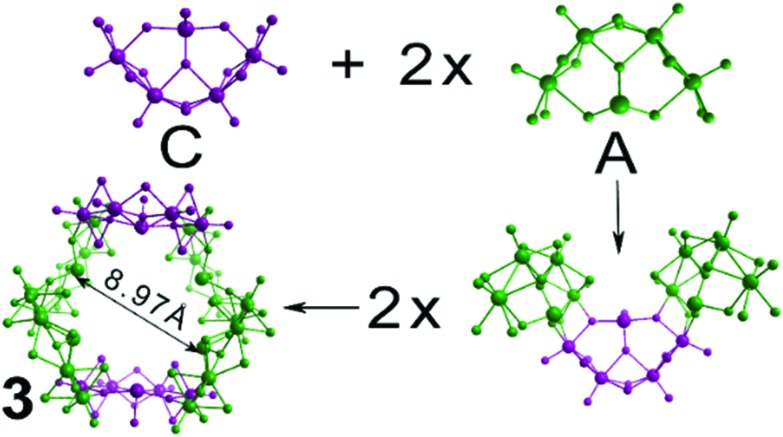
Structural representation of the building blocks (4 × **A** and 2 × **C**) used to construct cluster **3**. Cluster **3** exhibits a cavity of 8.97 Å occupied by K^+^ cations.

Compound **3**, [(Mo_2_O_2_S_2_)_12_(TeO_3_)_4_(TeO_4_)_2_(OH)_18_]^10–^, is the largest member of this family of clusters and is constructed from four type **A** and two type **C** building blocks. These building blocks form a large ring and are arranged in an alternating fashion, with each one rotated 180° relative to the two on either side of it. This gives rise to a structure reminiscent of the chair-conformer of cyclohexane. The central cavity of the molecule is 8.97 Å in diameter, with all lone pairs on the Te atoms pointing towards the centre of the cavity. Crystallographic studies revealed the presence of disordered electron density within the cavity attributable to the presence of potassium cations. The ring shaped topology in combination with the directionality of the tellurium based lone pair electrons makes a cluster framework that resembles an inorganic “crown ether” topology with the ability to bind different cations in its central cavity.

Even though the building blocks used to construct cluster **3** are considered relatively symmetric, the overall structure exhibits less symmetry elements than excepted due to the relevant orientation between the two **C**-type and four **A**-type building blocks. Thus, the point group of cluster **3** is lowered to C_S_, which exhibits only a reflection plane and involves the Te-atoms of the two **C**-type building blocks. Very subtle and localised changes in coordination geometry and orientation of the building blocks drastically reduced the symmetry of the entire molecule. It is worth noting at this point a few synthesis considerations which influence the formation of **1–3**. In each case, the reaction takes place under the same conditions. However, there is a narrow range of pH values (6.8–7.8) which distinguishes the different assembly processes (see the ESI†). Importantly, higher pH values (7.8) facilitate the formation of the Te-based dimers which are crucial for the templated formation of **2**.

In conclusion, we have discovered and characterised the first members of a new family of POTM clusters which are constructed using [Mo_2_O_2_S_2_]^2+^ dimers and templated solely by the tellurite anion. These clusters range in nuclearity from 8 Mo centres in the simple ring of compound **1** to 24 Mo centres in the inorganic “crown ether” analogue, compound **3**. The interaction of tellurite anions with the [Mo_2_O_2_S_2_]^2+^ dimer units gave rise to the formation of two fundamentally new building blocks, {(Mo_2_O_2_S_2_)_3_(Te_2_O_6_)(OH)_8_} **C** and {(Mo_2_O_2_S_2_)_3_(Te_2_O_6_)(OH)_8_} **D**. Interestingly, the ability of the tellurite anion to aggregate into small clusters, {Te_2_O_6_}, not only templated the formation but also induced asymmetry to the discovered building blocks and influenced further their connectivity and assembly. This resulted finally in the spontaneous assembly of the second example^30^ of POTM-based chiral cluster, **2**, and dramatically lowered the overall symmetry to *C*_S_ for **1** and **3**. In future work, we will attempt to generate new templates which will give rise to new building blocks and will also extend our symmetry breaking approach to gain access to new POTM structural features, nuclearities and symmetries.

## Conflicts of interest

The authors declare no conflicts of interest.

## Supplementary Material

Supplementary information

Crystal structure data
