# Supplementary material for: Spontaneous formation of a chiral (Mo2O2S2)2+-based cluster driven by dimeric {Te2O6}-based templates
Source: Dalton Trans. 2018 Mar 29;47(18):6283–7. doi: 10.1039/c8dt00832a (PMC5944425; doi:10.1039/c8dt00832a)
Supplement: Supplementary file 1 [file DT-047-C8DT00832A-s001.pdf]

Electronic Supplementary Material (ESI) for Dalton Transactions.
This journal is © The Royal Society of Chemistry 2018

Spontaneous Formation of Chiral $(Mo_2O_2S_2)^{2+}$-based Cluster Driven by Dimeric $\{Te_2O_6\}$ Based Templates

Jamie W. Purcell, De-Liang Long, Edward C. Lee, Leroy Cronin* and Haralampos N. Miras*

Supporting Information

Page S2: Experimental

Page S4: Infra-Red Spectroscopy

Page S5: Thermogravimetric Analysis

Page S7: ESI-Mass Spectrometry

Page S13: Crystallographic Data

Page S15: Additional References

Experimental

All reagents were purchased from Fisher Scientific, Sigma Aldrich and Alfa Aesar and used as provided with no further purification. The $[Mo_2O_2S_2]^{2+}$ dimeric unit was prepared according to the modified procedure published by Cadôt *et al* in 1998.[1]

X-Ray Crystallography: Data were collected at 150(2)K using a Bruker AXS Apex II [$\lambda(Mo_{K\alpha})$ = 0.71073 Å] equipped with a graphite monochromator.

Structures were solved using Direct methods with SHELXS-2013[2] and SHELXL[3] using WinGX routines.[4] Refinement was achieved by full-matrix least squares on F^2 via SHELXL-2013. Corrections for incident and diffracted beam absorption effects were applied using analytical methods.[5]

All data manipulation and presentation steps were performed using WinGX. Details of interest about the structure refinement are given in the tables at the end of this document.

The X-ray crystallographic data reported in this article have been deposited at the Crystallographic Data Centres. For compound **1** and **2** the data can be obtained from FIZ Karlsruhe, 76344 Eggenstein-Leopoldshafen, Germany (fax: (+49)7247-808-666; e-mail: crysdata@fiz-karlsruhe.de), on quoting the deposition number CSD-433070 and 433071. For compound **3**, the data can be obtained free of charge from the Cambridge Crystallographic Data Centre via www.ccdc.cam.ac.uk/data_request/cif under deposition number CCDC-1550063.

Fourier Transform Infra-Red Spectroscopy: Samples were prepared as KBr discs and FT-IR spectra were collected in transmission mode using a Shimadzu IRAffinity-1S Fourier Transform Infra-Red Spectrophotometer. Wavenumbers (ν) are given in cm^{-1}

Electrospray Ionisation Mass Spectrometry (ESI-MS) was performed on a Waters Synapt-G2 HDMS spectrometer operating in sensitivity mode, equipped with a quadropole and time of flight (Q-ToF) module for MS analysis. All samples were prepared by dissolving in 1:10 H_2O:CH_3CN (HPLC grade) to a concentration of *ca.* 1 x 10^{-5} M and injected directly at a flow rate of 5 µL min^{-1} using a *Harvard* syringe pump. All spectra were collected in negative ion mode and analysed using the *Waters* MassLynx v4.1 software.

For all measurements the following parameters were employed: capillary voltage: 2.5 kV; sample cone voltage: 10.0 V; extraction cone voltage: 4.0 V; source temperature: 80 ˚C; desolvation temperature: 180 ˚C; cone gas flow: 15 L h^{-1} (N_2); desolvation gas flow: 750 L h^{-1} (N_2).

Thermogravimetric Analysis: Analysis was performed on a TA Instruments Q500 Thermogravimetric Analyser under nitrogen flow with a typical heating rate of 10° C/min from room temperature up to 800° C, unless otherwise stated

Elemental Analysis: Mo, S and Te content were determined by ICP-OES analysis according to the following procedure: 5-10 mg sample material was digested by adding 1 mL deionised water and 2mL conc. HNO_3 to the sample in a digestion beaker. The sample solution was warmed until clear before being allowed to cool and a further 5mL deionised water added. The resulting solution was transferred quantitatively with washings to an A class 50mL volumetric flask and made up to the mark with deionised water. A blank sample was also prepared simultaneously to account for any

digestion interferences. The samples were transferred to 50mL polypropylene centrifuge tubes and analysed on an Agilent SVDV 5100 ICP-OES using the SVDV mode and appropriate calibration standards.

Carbon and nitrogen content was analysed by the University of Glasgow microanalysis service within the School of Chemistry.

Potassium content was determined using a Corning 410 Flame Photometer using the same samples and calibration standards used in the ICP-OES analysis.

Synthesis of $K_3[(Mo_2O_2S_2)_4(TeO_3)(OH)_9]\cdot20H_2O$, 1: Na_2TeO_3 (0.0501 g, 0.226 mmol) was dissolved in 20 mL distilled water to form a clear, colourless solution. Dimeric $[Mo_2O_2S_2]^{2+}$ (5 mL, 0.68 mmol) was added, upon which the colour of the solution transitioned from colourless, through red and cloudy yellow to black. 1M $K_2CO_{3(aq)}$ was used to bring the pH to 5.13, with the colour being a cloudy yellow. The reaction mixture was then stirred at room temperature for 1 hour during which time the pH rose to 6.87. The solution was filtered and kept at 18 °C and after 1 week orange, cubic crystals suitable for X-ray diffraction studies were collected (57.8 mg, 20.67 % based on Mo). Elemental Analysis of $H_{19}K_3Mo_8O_{25}S_8Te$ (FW: 1688.01): Theoretical (Found)%: Mo 45.46 (44.70), S 15.20 (16.43), Te 7.56 (8.23), K 6.95 (6.10).

Synthesis of $K_8[(Mo_2O_2S_2)_{10}(TeO_3)(Te_2O_6)_2(OH)_{18}]\cdot45H_2O$, 2: Na_2TeO_3 (0.05g, 0.226mmol) was dissolved in 20 mL distilled water to form a clear, colourless solution. Addition of 5 mL $[Mo_2O_2S_2]^{2+}$ (0.68 mmol) followed which resulted in a series of colour change from colourless to cloudy orange to black. The pH of the solution was adjusted to 5.48 with 1M K_2CO_3 after which the reaction mixture turned cloudy yellow. After stirring at room temperature for 1 hour, during which time the pH rose to 7.79, the reaction mixture was filtered and the resulting clear orange solution was stored at 5 °C for 1-2 weeks, after which 64.2 mg orange diamond-shaped plate crystals suitable for X-ray diffraction studies were collected (17.38 % yield based on Mo) Elemental Analysis of $H_{98}K_8Mo_{20}O_{93}S_{20}Te_5$ (FW: 5097.47): Theoretical (Found)%: Mo 37.64 (37.17), S 12.58 (13.16), Te 12.52 (12.80), K 6.14 (6.10).

Synthesis of $(NMe_4)K_9[(Mo_2O_2S_2)_{12}(TeO_3)_4(TeO_4)_2(OH)_{18}]\cdot48H_2O$, 3: Na_2TeO_3 (0.055 g, 0.248 mmol) was dissolved in 20 mL distilled water to form a clear, colourless solution. Addition of 5mL $[Mo_2O_2S_2]^{2+}$ (0.68mmol) followed to give a black colour to the solution. 1M K_2CO_3 was used to adjust the pH to 5.04 with the colour becoming cloudy orange. The reaction mixture was stirred at room temperature for 1 hour, during which time the pH rose to 6.97. The reaction mixture was filtered, resulting in a clear orange solution which was kept at 18 °C. Within one week, triangular orange plate crystals formed that were suitable for X-ray diffraction studies. 65.1 mg material was collected (23.04 % yield based on Mo) Elemental Analysis of $C_4H_{110}K_9Mo_{24}NO_{102}S_{24}Te_6$ (FW: 5994.30): Theoretical (Found)%: Mo 38.41 (38.11), S 12.84 (11.99), Te 12.77 (11.63), K 5.87 (6.03).

Infra-Red Spectroscopy

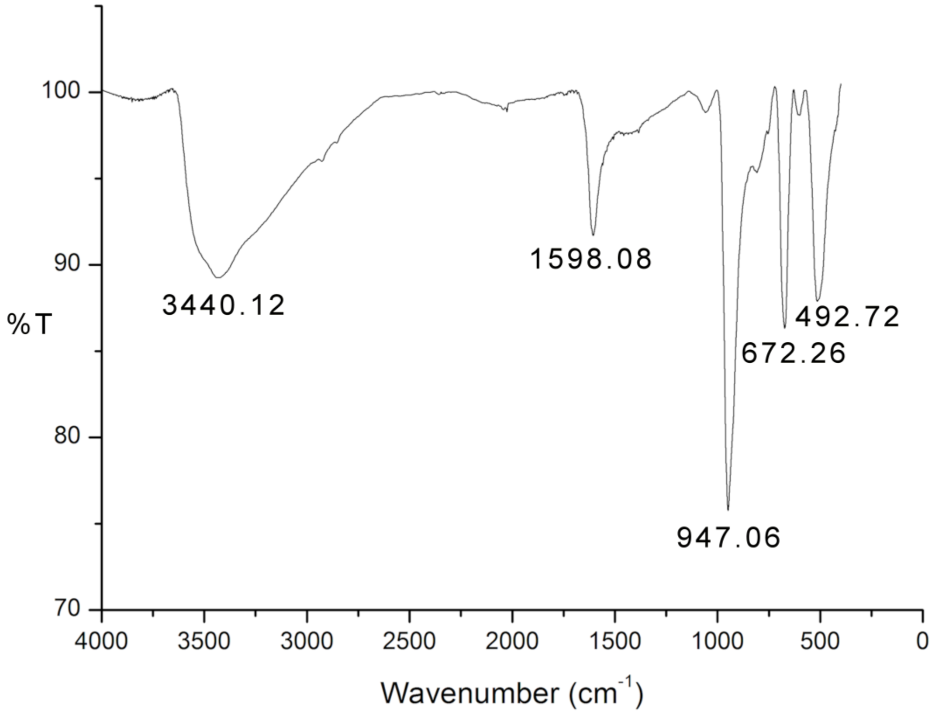

Figure S1: FT-IR spectrum of Compound **1**

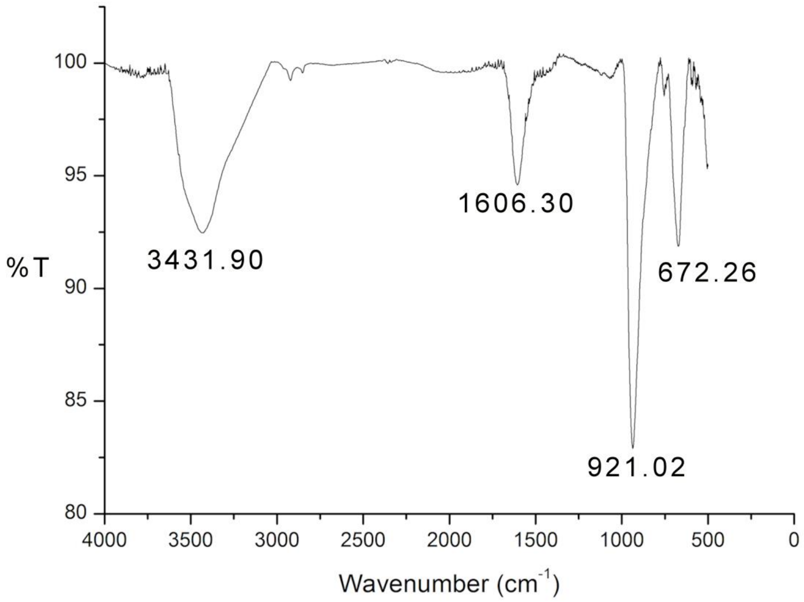

Figure S2: FT-IR spectrum of Compound **2**

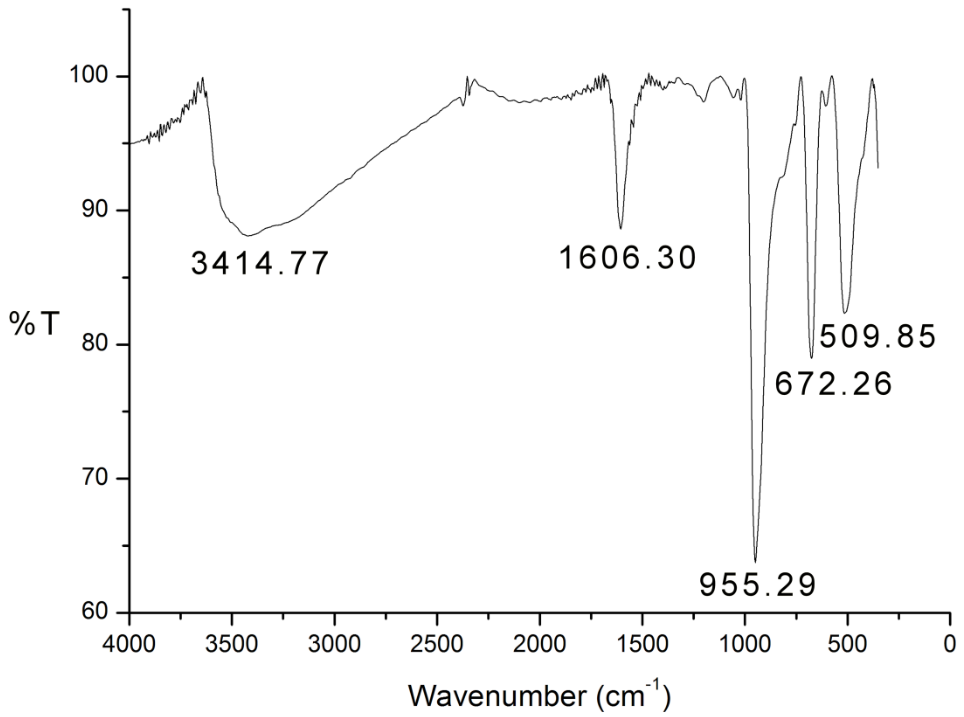

Figure S3: FT-IR Spectrum of Compound **3**

Thermogravimetric Analysis

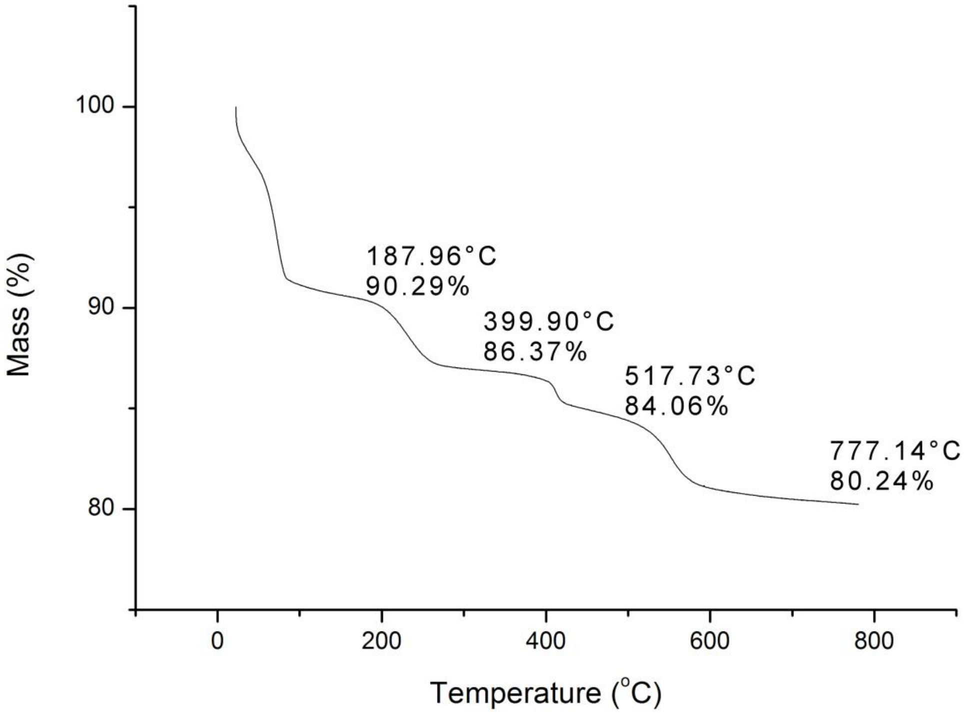

Figure S4: TGA trace of Compound **1**

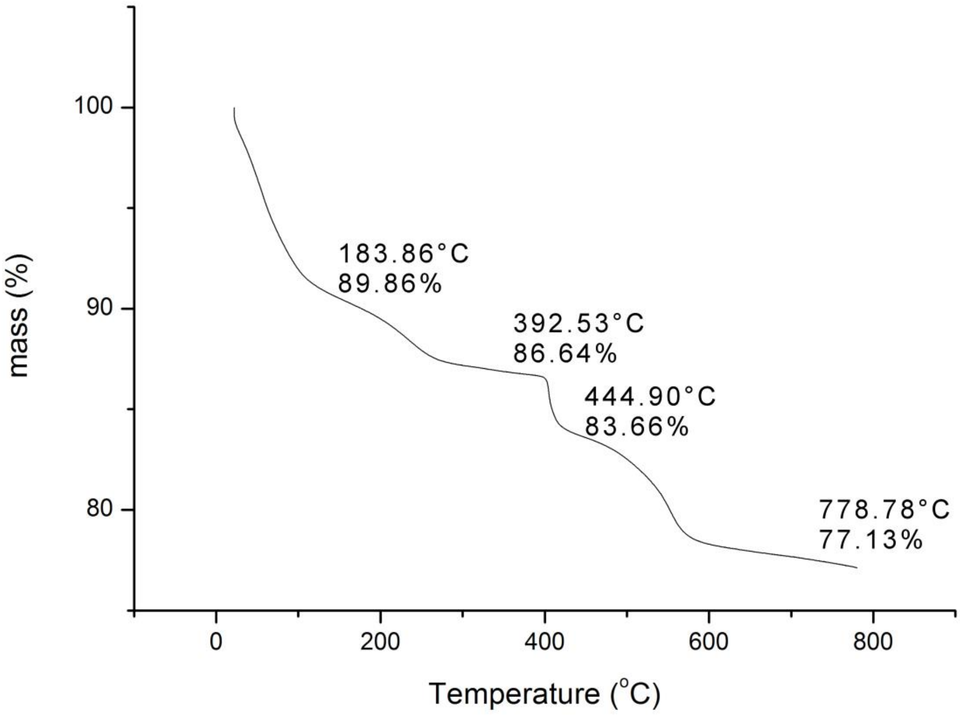

Figure S5: TGA trace of Compound **2**

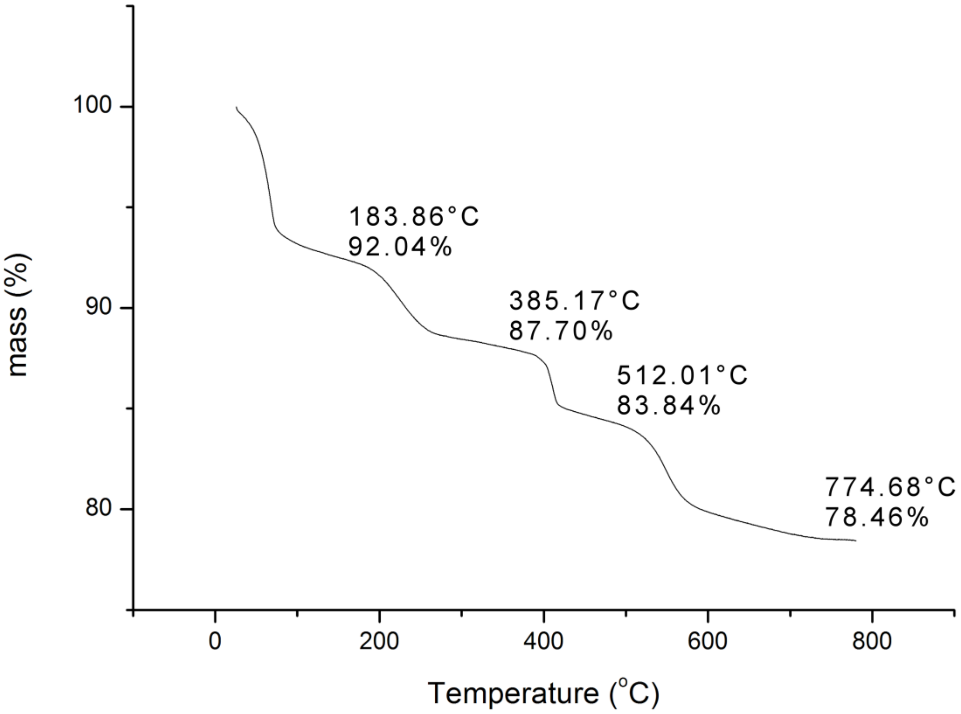

Figure S6: TGA trace of Compound **3**

ESI-Mass Spectrometry

We have employed electrospray ionisation mass spectrometry and performed the experiments in ion mobility mode in an effort to de-convolute and identify the species which correspond to the observed envelopes. Not only have we been able to use this technique to comment on the solution stability of each of the three compounds, we have also been able to confirm that they fragment into their fundamental building blocks which is indicative of the building block library stability.

Compound **1** revealed three envelopes tentatively assigned to the intact cluster centred at c.a. 1464.9, 1486.8 and 1502.8 m/z respectively (Table S1). In addition to this there are three further envelopes that we have assigned to the partially fragmented species derived from the loss of just one constituent part, such as a $[Mo_2O_2S_2]^{2+}$ dimeric unit or a tellurite anion. Interestingly, there are two additional envelopes that are large enough to correspond to ion pairing of molecular species centred at 1491.9 and 1496.2 m/z which correspond to the dimeric and trimeric aggregates of compound **1**.

Finally, it was possible to identify the type A virtual building block which is used to construct the cluster and gave an envelope centred at 802.3 m/z. The building block can fragment further through the loss of $[Mo_2O_2S_2]^{2+}$ dimeric units.

The mass spectrum of compound **2** suggests that this molecule is seemingly less stable than compound **1** under the employed ionization conditions (Table S2)**.** There are still two envelopes that were assigned to compound **2** after removal one of the three virtual building blocks (ca. 959.2 m/z) while the other having lost only a single tellurite template (ca. 1347.32 m/z). What is clearer from this spectrum is the presence of the constituent building blocks in this molecule. B type building blocks occur fairly regularly, as do C type building blocks- however these could equally be D type building blocks since C and D give identical m/z values.

The mass spectrum for compound **3** hasn't revealed any envelopes that could be assigned to the intact cluster indicating lack of stability, under the experimental conditions (Table S3). However we were able to detect virtual building blocks or bigger fragments of the cluster.

Interestingly we were able to observe a fragment that can be assigned to a species that is half of the cluster **3** and gives an envelope centered at 1467.9 m/z. This view is further reinforced by the presence of four envelopes that can be assigned to one third of the full molecule. Finally, two envelopes correspond to type A building blocks with an additional dimeric unit.

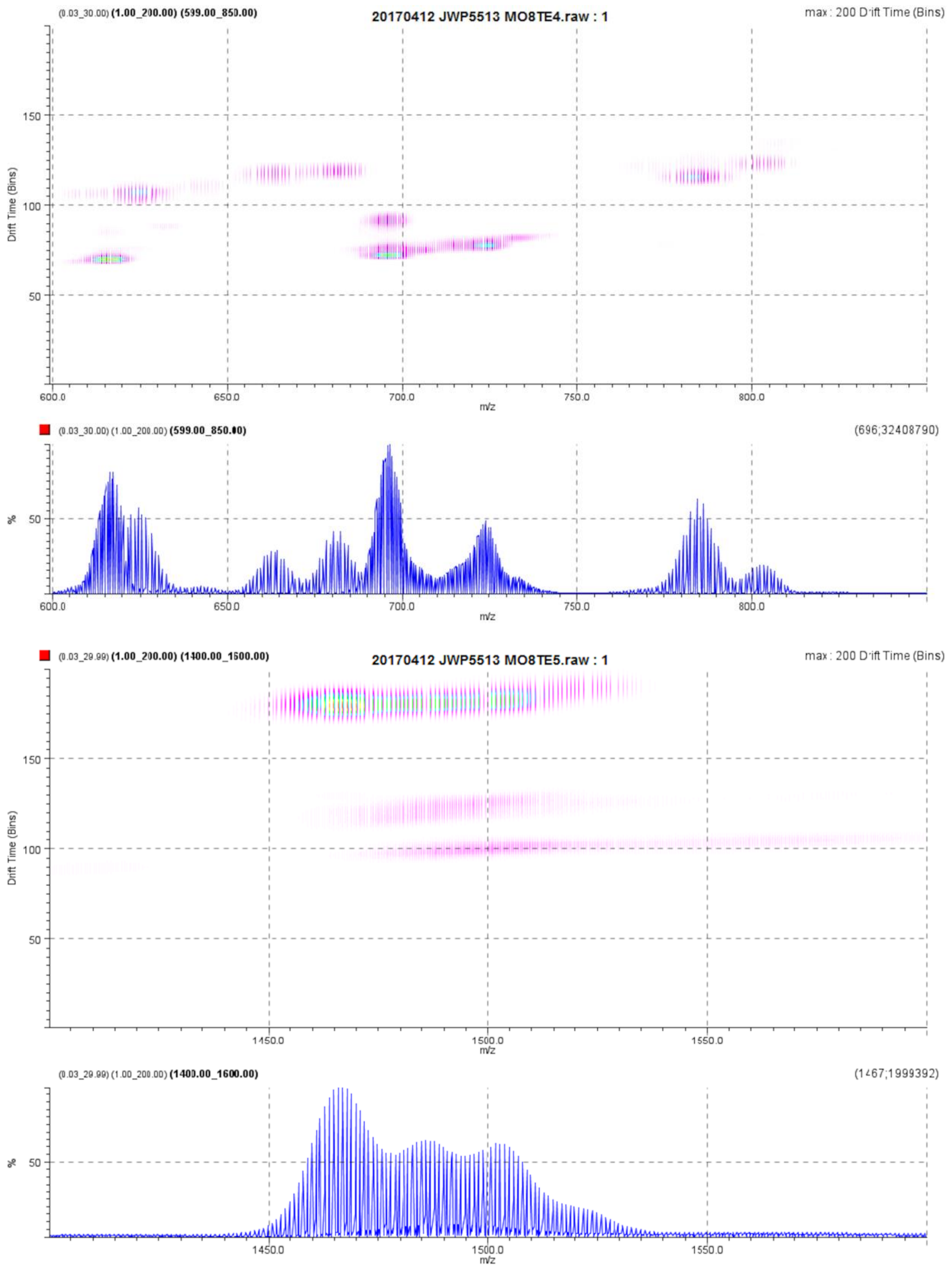

Figure S7: Ion Mobility ESI mass spectra for compound **1** (upper) and ESI mass spectra (lower)

Table S1: Ion Mobility Mass spectrometry peak assignments for Compound **1**

m/z	z	Assignment	Calculated m/z	Comment
616.48	-2	$K(Mo_2O_2S_2)_3(TeO_3)(OH)_7(H_2O)_2$	616.56	Full molecule – 1 dimer
625.50	-1	$(NMe_4)(Mo_2O_2S_2)(TeO_3)(OH)_2(H_2O)_3$	625.77	BB A – 1 dimer
663.44	-1	$(NMe_4)K(Mo_2O_2S_2)(TeO_3)(OH)_3(H_2O)_2$	663.72	BB A – 1 dimer
681.45	-1	$(NMe_4)K(Mo_2O_2S_2)(TeO_3)(OH)_3(H_2O)_3$	681.73	BB A – 1 dimer
695.43	-2	$(Mo_2O_2S_2)_3(TeO_3)_2(OH)_4(H_2O)_6$	695.54	BB A + TeO3 – 1 dimer
696.42	-2	$K(Mo_2O_2S_2)_3(TeO_3)_2(OH)_5(H_2O)_3$	696.51	Molecule + TeO3 – 1 dimer
724.41	-2	$(Mo_2O_2S_2)_4(OH)_{10}(H_2O)_7$	724.54	Full molecule – TeO3
784.38	-1	$K_4Na_2(Mo_2O_2S_2)(TeO_3)(OH)_7$	784.49	BB A – 1 dimer
802.39	-1	$(Mo_2O_2S_2)_2(TeO_3)(OH)_3$	802.39	BB A
1464.87	-1	$(Mo_2O_2S_2)_4(TeO_3)(OH)_7(H_2O)$	1464.90	Full molecule
1486.85	-1	$Na(Mo_2O_2S_2)_4(TeO_3)(OH)_8$	1486.88	Full molecule
1502.85	-1	$K(Mo_2O_2S_2)_4(TeO_3)(OH)_8$	1502.86	Full molecule
1491.85	-2	$(Mo_2O_2S_2)_8(TeO_3)_2(OH)_{14}(H_2O)_5$	1491.92	2 molecules
1496.18	-3	$K_2(Mo_2O_2S_2)_{12}(TeO_3)_3(OH)_{23}(H_2O)_2$	1496.21	3 molecules

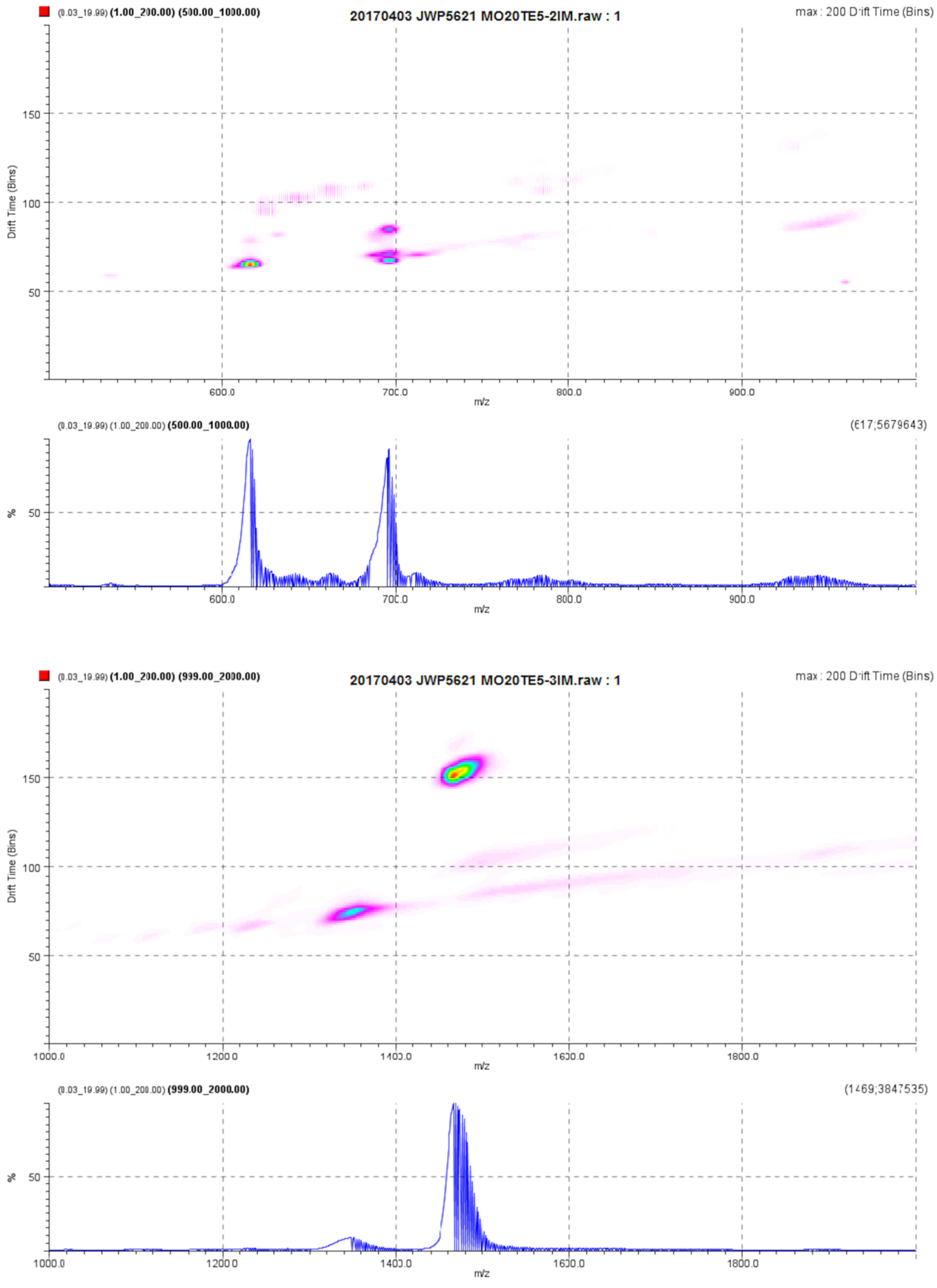

Figure S8: Ion Mobility ESI Mass spectra for Compound **2** (upper) and ESI mass spectra (lower)

Table S2: Ion Mobility mass spectrometry peak assignments for Compound **2**

m/z	z	Assignment	Calculated m/z	Comment
616.50	-2	$K(Mo_2O_2S_2)_3(TeO_3)(OH)_7(H_2O)_2$	616.56	BB B
625.51	-1	$(NMe_4)(Mo_2O_2S_2)(TeO_3)(OH)_2(H_2O)_3$	625.77	BB A – 1 dimer
643.51	-1	$(NMe_4)(Mo_2O_2S_2)(TeO_3)(OH)_2(H_2O)_4$	643.78	BB A – 1 dimer
663.47	-1	$(NMe_4)K(Mo_2O_2S_2)(TeO_3)(OH)_3(H_2O)_2$	663.72	BB A – 1 dimer
681.46	-1	$(NMe_4)K(Mo_2O_2S_2)(TeO_3)(OH)_3(H_2O)_3$	681.73	BB A – 1 dimer
695.45	-2	$(Mo_2O_2S_2)(TeO_3)_2(OH)_4(H_2O)_6$	695.54	BB C – 1 dimer
696.45	-2	$K(Mo_2O_2S_2)_3(TeO_3)_2(OH)_5(H_2O)_3$	696.51	BB C
696.94	-2	$K_2(Mo_2O_2S_2)_3(OH)_6(TeO_3)_2$	697.48	BB C
712.45	-2	$K_4Na(Mo_2O_2S_2)_3(TeO_3)(OH)_{11}(H_2O)$	712.51	BB B
945.29	-2	$(Mo_2O_2S_2)_4(TeO_3)_3(OH)_4(H_2O)_8$	945.37	BB C + {TeO_3}
959.43	-3	$K_2(Mo_2O_2S_2)_7(TeO_3)_3(OH)_{13}(H_2O)_2$	959.29	Molecule – BB C
1348.03	-3	$K_3(Mo_2O_2S_2)_{10}(TeO_3)_4(OH)_{18}(H_2O)_2$	1347.32	Full Molecule- 1 {TeO_3}
1466.95	-1	$K(Mo_2O_2S_2)_3(TeO_3)_2(OH)_4(H_2O)_8$	1466.07	BB C

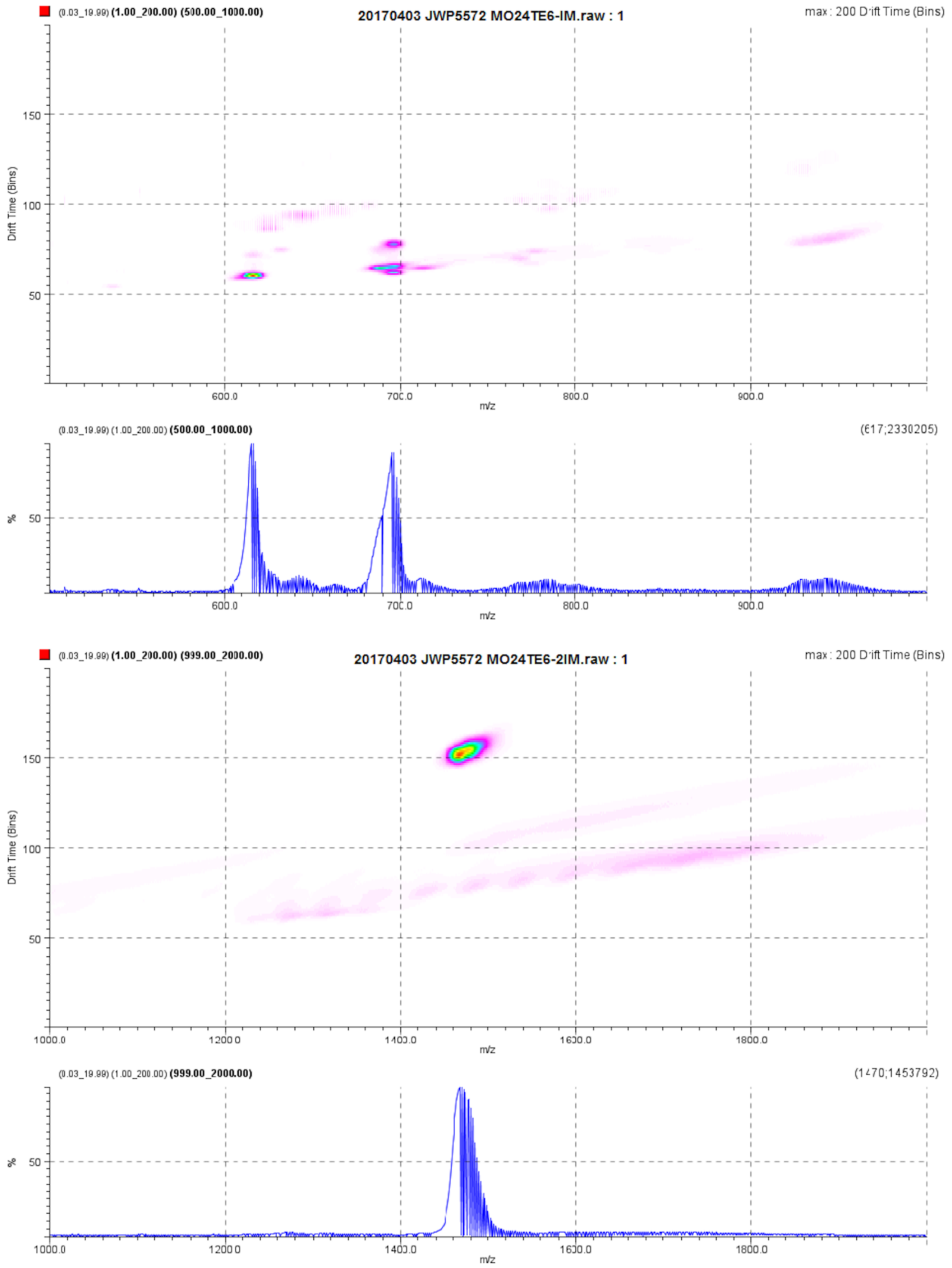

Figure S9: Ion Mobility ESI mass spectra for compound **3** (upper) and ESI mass spectra (lower)

Table S3: Ion Mobility ESI mass spectrometry peak assignments for compound **3**

m/z	z	Assignment	Calculated m/z	Comment
616.02	-2	$(Mo_2O_2S_2)_3(TeO_3)(OH)_6(H_2O)_5$	615.60	BB A + 1 dimer
625.52	-1	$(NMe_4)(Mo_2O_2S_2)(TeO_3)(OH)_2(H_2O)_3$	625.77	BB A – 1 dimer
643.52	-1	$(NMe_4)(Mo_2O_2S_2)(TeO_3)(OH)_2(H_2O)_4$	643.78	BB A – 1 dimer
664.49	-1	$K_2(Mo_2O_2S_2)(TeO_3)(OH)_3(H_2O)_4$	664.61	BB A – 1 dimer
689.45	-2	$KNa(Mo_2O_2S_2)_3(TeO_3)_2(OH)_6$	689.49	2x BB A – 1 dimer
696.46	-2	$K(Mo_2O_2S_2)_3(TeO_3)_2(OH)_5(H_2O)_3$	696.51	2x BB A – 1 dimer
776.30	-2	$(Mo_2O_2S_2)_4(TeO_3)(OH)_8(H_2O)_5$	776.97	2x BB A - $\{TeO_3\}$
767.89	-2	$(Mo_2O_2S_2)_4(TeO_3)(OH)_8(H_2O)_4$	767.97	2x BB A - $\{TeO_3\}$
946.30	-2	$K_2(Mo_2O_2S_2)_3(TeO_3)(OH)_8(H_2O)_2$	645.55	BB A + 1 dimer
1467.98	-2	$K_2(Mo_2O_2S_2)_6(Mo^{IV}Mo^{V}O_2S_2)H(TeO_3)_3(OH)_{12}(H_2O)_6$	1466.96	Half-Molecule + 1 dimer

Crystallography Data

Table S4: Crystallographic Data for Compound **1**

Identification code	JWP3361
Empirical formula	H29 K3 Mo8 O30 S8 Te
Formula weight	1778.13
Temperature	150(2)K
Wavelength	0.71073Å
Crystal system, space group	Monoclinic, P 21/c
Unit cell dimensions	a=10.0033(8)Å α=90°<br>b= 12.4181(11)Å β=100.349(4)°<br>c=15.4775(13)Å γ=90°
Volume	1891.4(3)Å^{3}
Z, Calculated density	1, 3.122 Mg/m^{3}
Absorption coefficient	4.183mm^{-1}
F(000)	1684
Crystal size	0.100 x 0.080 x 0.060 mm^{3}
θ range for data collection	2.641 to 25.998°
Limiting indices	-12<=h<=12, -13<=k<=15, -15<=l<=19
Reflections collected / unique	13784/ 3692
Completeness to θ = 25.242	99.4%
Absorption correction	Empirical
Max. and Min. transmission	0.745 and 0.426
Refinement method	Full-Matrix Least Squares on F^{2}
Data / restraints / parameters	3692/ 0/ 256
Goodness-of-fit on F^{2}	1.220
R indices (all data)	R1=0.0613, wR2=0.1314
Extinction coefficient	0.00085(15)
Largest diff. peak and hole	1.42 and -1.79 e.Å^{-3}

Table S5: Crystallographic Data for Compound **2**

Identification code	JWP3422
Empirical formula	H108 K8 Mo20 O98 S20 Te5
Formula weight	5187.66
Temperature	150(2)K
Wavelength	0.71073Å
Crystal system, space group	Triclinic, $P\overline{1}$
Unit cell dimensions	a=15.2433(10)Å α=107.582(4)°<br>b=18.1235(12)Å β=95.258(4)°<br>c=25.9548(18)Å γ= 108.882(3)°
Volume	6323.3(8)$Å^3$
Z, Calculated density	2, 2.725Mg/m^3
Absorption coefficient	3.735mm^{-1}
F(000)	4928
Crystal size	0.100 x 0.080 x 0.070 mm^3
θ range for data collection	1.675 to 25.999°
Limiting indices	-18<=h<=19, -20<=k<=22, -32<=l<=32
Reflections collected / unique	91451/ 24762
Completeness to θ = 25.242	99.7%
Absorption correction	Empirical
Refinement method	Full Matrix Least Squares on F^2
Data / restraints / parameters	24762/0/1366
Goodness-of-fit on F^2	1.032
R indices (all data)	R1=0.0785, wR2=0.1492
Extinction coefficient	0.00019(3)
Largest diff. peak and hole	2.46 and -2.48 e.$Å^{-3}$

Table S6: Crystallographic Data for Compound **3**

Identification code	ECL3832
Empirical formula	C4 H126 N K9 Mo24 O110 S24 Te6
Formula weight	613.55
Temperature	150(2)K
Wavelength	0.71073Å
Crystal system, space group	Monoclinic, C 2/c
Unit cell dimensions	a=33.906(3)Å α=90°<br>b=18.8942(16)Å β=119.541(4)°<br>c=29.641(3)Å γ=90°
Volume	16520(3)$Å^3$
Z, Calculated density	4, 2.468 Mg/m^3
Absorption coefficient	3.412mm^{-1}
F(000)	11648
Crystal size	0.100 x 0.080 x 0.070 mm^3
θ range for data collection	1.673 to 26.000°
Limiting indices	-41<=h<=41, -23<=k<=23, -36<=l<=36
Reflections collected / unique	120222/ 16217
Completeness to θ = 25.242	99.9%
Absorption correction	Empirical
Refinement method	Full Matrix Least Squares on F^2
Data / restraints / parameters	16217/ 0/ 848
Goodness-of-fit on F^2	1.025
R indices (all data)	R1=0.0847, wR2=0.1562
Extinction coefficient	0.000038(5)
Largest diff. peak and hole	2.73 and -1.18 e.$Å^{-3}$

References

1 E. Cadot, B. Salignac, S. Halut and F. Sécheresse, *Angew. Chem. Int. Ed.*, **1998**, *37*, 611-613

2 G. Sheldrick, *Acta. Cryst. A*, **1990**, *46*, 467-473

3 G. Sheldrick, *Acta. Cryst. A*, **2008**, *64*, 112-122

4 L. Farrugia, *J. Appl. Crystallogr.*, **1999**, *32*, 837-838

5 R. C. Clark and J. S. Reid, *Acta. Cryst. A*, **1995**, *51*, 887-897
